# Bone peg fixation of a large chondral fragment in the weight-bearing portion of the lateral femoral condyle in an adolescent: a case report

**DOI:** 10.1186/1752-1947-8-316

**Published:** 2014-09-23

**Authors:** Hiroshi Nakayama, Shinichi Yoshiya

**Affiliations:** 1 Department of Orthopaedic Surgery, Hyogo College of Medicine, 1-1Mukogawa-cho, Nishinomiya, Hyogo 663-8501, Japan

**Keywords:** Bone peg, Chondral fracture, Femoral condyle

## Abstract

**Introduction:**

Chondral fracture of the knee is relatively rare and the optimal treatment option for this injury is still controversial. In this report, we present the case of a patient with this injury who was treated surgically using the bone peg fixation procedure. There has been no literature reporting the use of this technique for fixation of a detached chondral fragment.

**Case presentation:**

The patient was a 14-year-old Japanese boy who sustained a knee injury while kicking a soccer ball. Although routine radiographs showed no abnormality, magnetic resonance imaging showed a large full-thickness chondral defect in the weight-bearing portion of his lateral femoral condyle and a detached chondral fragment in the anterior region. The size of the defect (fragment) was 2cm by 1.5cm. At surgery, the chondral fragment was fixed with eight cortical bone pegs that were harvested from the anteromedial aspect of his tibia.

**Conclusions:**

The postoperative magnetic resonance imaging at 4 months and the second-look arthroscopy at 12 months revealed apparent healing of the fragment. In the final follow-up examination at 26 months, a physical examination showed no swelling with recovery of full range of motion, and he could play soccer at the pre-injury level with no complaint. Based on the clinical course of this patient, it is thought that bone peg fixation can be a valuable option for fixation of a large chondral fracture of the knee.

## Introduction

Chondral fracture of the femoral condyle is relatively rare and predominantly encountered as a sports-related injury in adolescence due to inferior mechanical properties of the bone–cartilage junction in this age range [1-3]. Impact of the patellar facet or tibial plateau against the femoral condyle during a twisting type of injury is thought to be an injury mechanism [2-6]. Since chondral fragments do not show up in routine radiographs, this injury may not be correctly diagnosed at the initial presentation; however, careful assessment of magnetic resonance images (MRI) can reveal chondral loose bodies as well as full-thickness defects on the articular surface. This injury often extends over a large area, and sometimes involves the weight-bearing portion [4-6]. For large lesions in the weight-bearing portion, restoration of cartilaginous integrity by internal fixation of the chondral fragment should be considered a treatment option. Regarding the surgical procedure, metal and bioabsorbable devices have been utilized to fix the intra-articular fragment in previous studies [2,4-7]; however, a metal device requires subsequent removal in a secondary surgery, while use of a bioabsorbable material within the joint may induce a foreign body reaction in the process of degradation and reabsorption [8-10]. As an alternative option, bone peg fixation of the intra-articular osteochondral fragment has been reported with favorable results in patients with osteochondritis dissecans [11-13]. Use of the bone peg affords rigid fixation and may provide an additional advantage of biological healing enhancement; however, there has been no literature reporting the use of this technique for fixation of a detached chondral fragment.

In this report, we present the clinical characteristics and outcome of a patient with a chondral fracture in the weight-bearing region of his lateral femoral condyle who was successfully treated with bone peg fixation.

## Case presentation

**Figure 1:**
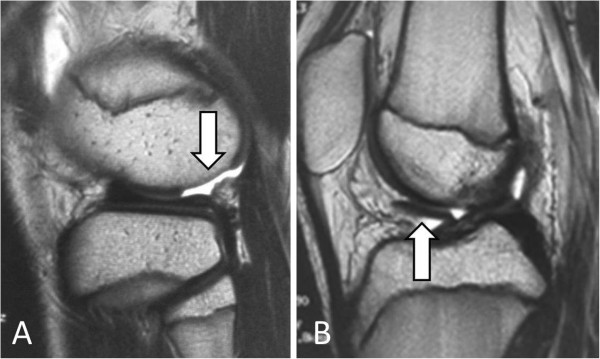
**T2-weighed sagittal magnetic resonance images at the initial visit. (A)** A full-thickness chondral defect in the weight-bearing portion of the lateral femoral condyle (white arrow). **(B)** A large chondral fragment in the anterior joint space (white arrow).

**Figure 2:**
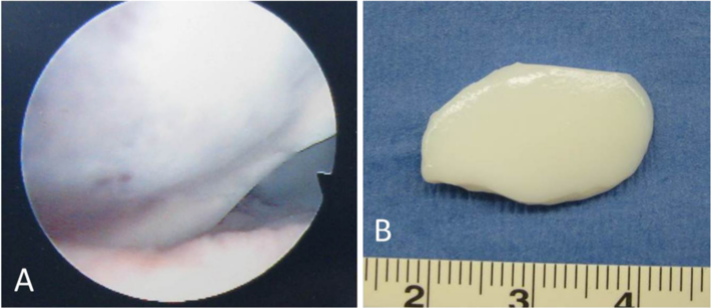
**Intraoperative findings. (A)** Arthroscopic view of the full-thickness chondral defect of the lateral femoral condyle. **(B)** Macroscopic appearance of the chondral fragment (2cm × 1.5cm in size).

**Figure 3:**
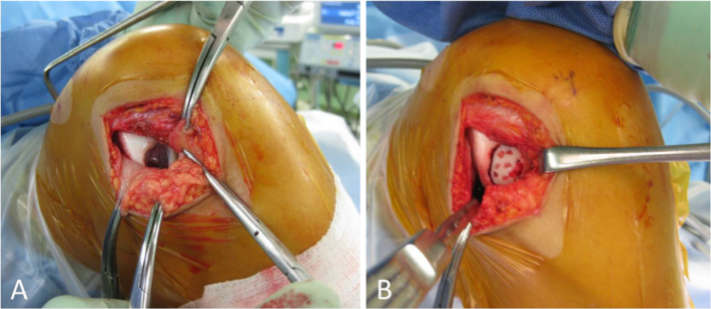
**Intraoperative photographs showing the surgical procedure. (A)** Removal of thin tissue covering the surface of the chondral defect. **(B)** Fixation of the fragment using eight bone pegs.

The review board of our institute approved this study, and an appropriate written informed consent was obtained from the patient and his family. A 14-year-old Japanese boy presented with pain and swelling in his right knee. He sustained an injury a month ago while kicking a soccer ball with his left leg. Following the injury, he first consulted a local clinic and was then referred to our hospital due to persistent symptoms.At his initial visit to our hospital, a physical examination showed mild effusion and limited extension without signs of ligamentous or meniscal injuries. A radiographic examination showed no abnormal findings such as fracture and malalignment. However, MRI revealed a large, full-thickness chondral defect in the weight-bearing portion of his lateral femoral condyle and a detached fragment with a length of 2cm in the anterior region (Figure 1). No other findings indicating bony or soft tissue injuries were detected on MRI. Based on these clinical and image findings, a diagnosis was made for detached chondral fracture of the lateral femoral condyle. Considering the size and location (weight-bearing area) of the injury, open reduction and internal fixation of the fragment was selected as a treatment option.Surgery was performed 6 weeks after the injury. Arthroscopic examination prior to arthrotomy showed a large, full-thickness chondral defect of his lateral femoral condyle as well as a chondral fragment of corresponding size (Figure 2A). There were no other intra-articular injuries found in arthroscopy. Thereafter, lateral parapatellar arthrotomy was performed. The size of the loose body was 2cm by 1.5cm and no bony portion was attached to the fragment (Figure 2B). Subsequently, eight cortical bone pegs (each 2mm in diameter and 15mm in length) were harvested from the anteromedial aspect of the proximal tibia. Following the removal of the thin tissue covering the surface of his chondral defect (Figure 3A), the chondral fragment was fixed back to his subchondral bone using the bone pegs. The fragment fit into the defect well and an apparently smooth articular surface was restored with firm fixation achieved (Figure 3B).

**Figure 4:**
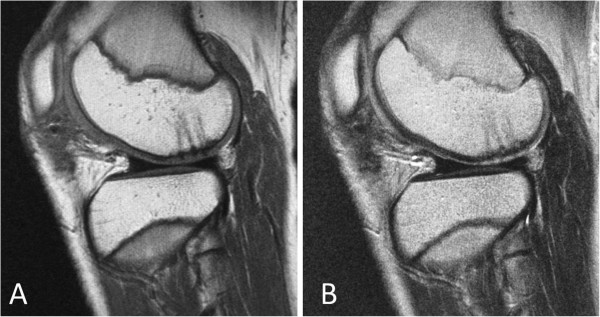
**Postoperative magnetic resonance images at 4 months showing apparent healing of the fixed fragment. (A)** T1-weighted sagittal image. **(B)** T2-weighted sagittal image.

**Figure 5:**
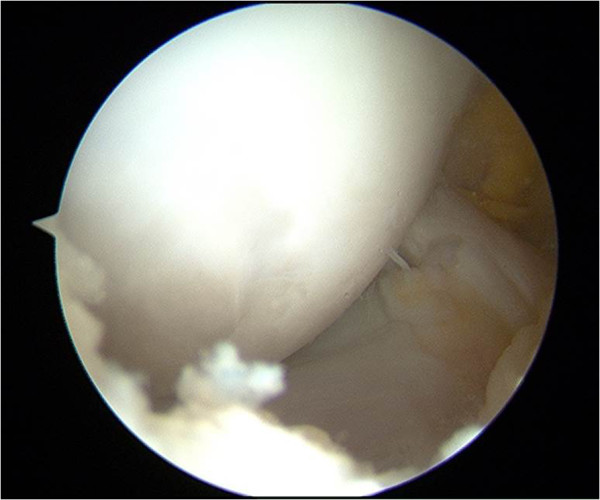
**Second-look arthroscopy at 12 months showed a smooth articular surface with a shallow hollow at the margin.** The head of the bone peg was no longer visible.

Postoperatively, his operated knee was immobilized in a cylinder cast for 3 weeks and passive range of motion was started thereafter. Partial weight bearing was allowed at 5 weeks with progression to full weight bearing at 8 weeks. Considering the location of the chondral fracture (contact position at flexion), use of a hinged brace with an extension blocking mechanism was instructed while walking to avoid the application of excessive load to the fixed fragment.The postoperative MRI performed at 4 months showed continuity of the articular surface, which suggested the progression of healing with maintenance of reduction (Figure 4). After confirmation of the apparent healing on MRI, he began jogging at 5 months and return to strenuous sport activities was permitted at 7 months. In order to directly confirm the healing status of the fixed fragment, a second-look arthroscopy was performed at 12 months. In the arthroscopic examination, the chondral fragment appeared firmly united to the underlying bone with restoration of a smooth articular surface (Figure 5). The patient could play soccer at a pre-injury level with no complaint at the final follow-up visit at 26 months.

## Discussion

Chondral fracture of the knee is a relatively uncommon injury. In 1985, Hopkinson *et al.*[2] reviewed their patient records and reported that chondral fractures were identified in eight of the 1095 knees (0.73%) arthroscoped at their institute. In accordance with the routine use of MRI as a part of the examination for knees with sports injuries, diagnostic accuracy for chondral injury has been improved and the incidence of this injury in our current practice is thought to be higher than the value in previous reports.

Regarding the injury mechanism of chondral fractures, Flachsmann *et al*. [1] conducted a biomechanical study using bovine cartilage bone laminates. They compared the strength of the osteochondral junction among the different maturation stages and showed that adolescent tissue exhibited significantly reduced fracture toughness under shear loading. Therefore, shear loading at the articular contact during a twisting injury is thought to be a predominant injury mechanism. Since the chondral defect in the reported case was located at the mid-lateral articular edge, patella-femoral contact during shifting motion of the patella may have been a mechanism of the injury. Mashoof *et al.*[4] reported seven cases with osteochondral injuries to this location that were associated with patella dislocation.

Chondral fractures with detached fragments require surgical intervention. Surgical options include removal and fixation of the fragment. Considering the importance of cartilage integrity, fixation of the fragment is a preferable option for a large lesion in the weight-bearing portion. Conventionally, a metal fixation device was used to fix the fragment [3]; however, use of metal devices requires reoperation for hardware removal. In recent relevant reports, bioabsorbable pins have become a principal option. Walsh *et al.*[6] reported the use of polyglycolic acid rods for fixation with successful outcomes in a majority of the cases. Nakamura *et al.*[7] reported a case with fixation of the fragment using poly-L-lactic acid (PLLA) pins. In this patient, complete healing at the osteochondral junction was accomplished as confirmed by histological examination. Although these reports showed a satisfactory outcome without procedure-related complications, postoperative complications such as foreign body reaction have been reported with the use of bioabsorbable fixation devices in the knee [8-10]. Moreover, chondral injury on the articular surface and hardware breakage have also been reported as other potential complications [14,15]. Bone pegs have been successfully used to fix osteochondral dissecans lesions and osteochondral fractures [11-13]. This method of fixation has several advantages over other fixation methods. First, secondary surgery for hardware removal is not required. Secondly, complications such as foreign body reaction and chondral injury can be avoided. Finally, insertion of autogenous bone through the interface may biologically enhance the healing process.

In the presented case, conditions for healing were not ideal because of the 6-week time period between injury and surgery and the lack of bony attachment to the fragment. Nevertheless, satisfactory healing was attained as confirmed by the postoperative MRI and the second-look arthroscopy. The use of bone peg as a fixation device in this case may have provided a favorable environment for healing both in a biomechanical and a biological aspect. However, this is a report of only one patient without histological confirmation of healing at the osteochondral junction. Moreover, the follow-up period is still short. Accumulation of further experiences for additional cases and longer term follow-up results are required to prove the efficacy of this procedure.

## Conclusions

Bone peg fixation procedure for a chondral fracture of the knee has not been reported in the previous literature. We utilized this fixation method for a patient with a large chondral fracture in the weight-bearing portion of the lateral femoral condyle. Healing at the fracture site was confirmed by MRI and second-look arthroscopy with a satisfactory functional outcome. Thus, it is thought that bone peg fixation can be a good option in surgical treatment of chondral fractures.

## Consent

Written informed consent was obtained from the patient’s parent for publication of this case report and accompanying images. A copy of the written consent is available for review by the Editor-in-Chief of this journal.

## Competing interests

The authors declare that they have no competing interests.

## Authors’ contributions

HN was a major contributor in writing the manuscript. HN performed the surgery and several examinations of the patient and carried out the follow-up of the patient. SY conceived of the study, participated in its design and coordination, and helped to draft the manuscript. Both authors read and approved the final manuscript.
